# Editorial: Biology and clinical applications of adipose-derived cells for skeletal regeneration

**DOI:** 10.3389/fbioe.2023.1221444

**Published:** 2023-05-23

**Authors:** Danièle Noël, Arnaud Scherberich

**Affiliations:** ^1^ IRMB, University of Montpellier, INSERM, Montpellier, France; ^2^ Bone Regeneration, Department of Biomedicine, University Hospital Basel, University of Basel, Basel, Switzerland; ^3^ Department of Biomedical Engineering, University of Basel, Basel, Switzerland

**Keywords:** adipose derived mesenchymal cells, endochondral ossification, cell characterization, extracellular vesicles, osteogenic differentiation, stromal vascular tissue, chondrogenic differentiation

Adipose-derived cells is a very generic term to refer to the various cell types, which can be obtained from a biopsy of human adipose tissue. Starting from a liposuction sample or a solid resection of adipose, it most commonly involves a step of cell isolation based on either enzymatic digestion or mechanical disruption, and sometimes even a combination thereof (Tiryaki et al., 2022). The enzymatic digestion of adipose tissue leads, after centrifugation to discard floating adipocytes, to a pellet of cells called the Stromal Vascular Fraction (SVF). SVF is a complex mixture of vascular endothelial progenitors and mesenchymal stromal progenitor cells, hematopoietic cells of various nature, preadipocytes, fibroblasts, smooth muscle cells and others yet to be fully characterized (Ramakrishnan and Boyd, 2018). Upon seeding of SVF cells onto tissue culture plastic and subsequent culture as monolayer, mesenchymal stromal cells present in SVF, referred to as adipose-derived mesenchymal stromal cells (ASC) attach and proliferate very similarly to bone marrow mesenchymal stromal cells (MSC), leading to a very strong enrichment in ASC already after the very first step of expansion (i.e., before passaging cells for the first time) (Müller et al., 2009). After this initial stage of expansion, ASC, characterized as CD73^+^, CD90^+^, CD44^+^, CD34^+^ cells, account for >70–80% of the expanded cells, reaching >95% at the end of the passage 1 (Bony et al., 2016). The remaining cells are mostly composed of vascular endothelial cells, important for some of their functional properties (Guerrero et al., 2022).

The mechanical dissociation of adipose results in a suspension of small pieces of body fat tissue, containing SVF cells and extracellular matrix (ECM), with or without maintenance of viable adipocytes. It is then called either Coleman Fat, microfat or nanofat (Ding et al., 2022). The term nanofat is however inappropriate since the resulting tissue is far from nanometric dimension by nature. A recent discussion at the 7th International Congress on Adipose Stem cell Treatments (AIRMESS/iCAST 2022) held at the Nescens Clinic in Génoliers, Switzerland, on 3 December 2022, suggested to define such fractionated adipose as Stromal Vascular Tissue (SVT) (https://sscf.ch/icast/icast2022/). Whatever the denomination and way of generating it, such SVT has been shown to be very helpful for various clinical applications (Ding et al., 2022). A better characterization of SVT is however of importance to account for and predict its quality and regenerative potential. In the present Research Topic, Claudia Cicione and colleagues compared different techniques to generate SVT and showed that this might have a very important effect on the stemness properties of ASC and the clinical potential of SVT.

It is important to note, in the context of the present Research Topic, that ASC, unlike bone marrow-derived MSC, are non-skeletal MSC by nature. There is however a plethora of evidence and reports since almost 2 decades showing that adipose-derived cells can be switched to genuine skeletal cells and be used for skeletal regeneration. SVF cells and monolayer-expanded ASC can become chondrogenic or osteogenic cells when combined with scaffolding materials, as reviewed in this Research Topic by Gohar Rahman and colleagues. A practical example of that is the fact that ASC-derived extracellular vesicles (EV) can induce osteogenic differentiation of ASC in graphene porous titanium alloy scaffolds, as shown by Xu Sun and colleagues. Another example is the following: Chen Cheng and colleagues show that ASC grown directly inside SVT *in vitro* and differentiated into hypertrophic chondrocytes are able to generate bone by endochondral ossification and repair a mandibular defect in rat. There is no doubt that bone regeneration with adipose-derived cells, thanks to a minimally invasive sourcing and a relevant abundancy, will continue to raise interest and progress in the future.
